# New perspectives on World Heritage management in the GCC legislation

**DOI:** 10.1186/s43238-022-00056-y

**Published:** 2022-06-22

**Authors:** Michał M. Wosiński

**Affiliations:** 1 Principal Archaeology and Cultural Heritage Specialist, Jacobs, Cracow, Poland; 2 ICOMOS Bahrain, Manama, Bahrain; 3 ICOMOS ICLAFI, Manama, Bahrain

**Keywords:** Heritage management, Heritage law, Antiquities law, Arab States, GCC

## Abstract

New legislative acts on heritage protection have been published in several Arab Countries in the last decade. Whilst this is a positive trend in general, what remains to be seen is the factual impact of the new legislation on the pressing issues of heritage management, including the holistic and integrated approaches, monitoring, enforcement and others. This paper compares the new legislative solutions from Gulf Arab Countries to the older ones and determines whether the new trends have potential of improving the heritage management systems. The findings of this paper are intended to increase the awareness of the still-marginal issue of heritage management in the heritage legislation of Arab region and promote the successful measures among the countries with outdated statutory heritage protection.

## Introduction

Inadequate regulatory framework is one of the main factors hindering the preservation of cultural heritage. Lack of provisions establishing appropriate mechanisms for protection and management and lack of their procedural integration with stakeholders of the planning processes undermines the efforts to safeguard the Outstanding Universal Value of the World Heritage properties in the Arab Region.

In its decisions, the World Heritage Committee often highlights the urgent need to better address the issue of an inadequate legislation and management by the States Parties from the Arab Region.^1^

Third Cycle of Periodic Reporting exercise for the Arab Region has been finalised in 2020. Based on its findings a Draft Action Plan^2^ was elaborated which summarises the identified challenges in the Arab Region and recommends areas for intensified focus. According to the Draft Action Plan (p. 3): ‘Many States Parties have made major updates to their national legislations for heritage incorporating many provisions of the World Heritage Convention and its application into their principal legislation. Yet, the incorporation of additional conventions, policies and programs will require further efforts in establishing policies, legislation, regulations and operational mechanisms. Legislations were noted as partially adequate by the majority of States Parties, and existing capacity/resources to enforce the legal framework could be strengthened.’ Referring to the legal protection and management, the Draft Action Plan indicates that (p. 6) ‘Legal frameworks for the protection of boundaries is considered adequate for only half of the sites, while for the buffer zones it is much less (…) Management systems are considered adequate for less than half of the World Heritage sites, and in more than half of the sites, a management system is only partially implemented. Statutory management plans or zoning plans are widespread, but there are deficiencies in other management tools related to visitors and tourism, environment and climate change, risk mitigation, and sustainable development.’ And finally, when referring to policy of World Heritage Properties, the Draft Action Plan states (p. 4) ‘The main issues that have been identified are in relation to insufficient availability of effective regulatory frameworks that require impact assessments for programmes or development projects.’

Considering the Committee decisions and findings of the Draft Action Plan there is a direct relation between the strong, up-to-date legal framework and the adequate management system for the World Heritage sites. The following pages will investigate this relation through establishing a reference for heritage management and analysing heritage law in six Gulf Cooperation Council (hereinafter: GCC) countries: Bahrain, Kuwait, Oman, Qatar, Saudi Arabia and United Arab Emirates (Sharjah).

## Part I. An adequate heritage management system

### Framework of Reference

The most comprehensive system of heritage management, applicable worldwide has been established by the World Heritage doctrine over the last five decades, since the entry into force of the 1972 Convention Concerning the Protection of the World Cultural and Natural Heritage (hereinafter: 1972 Convention). The management of cultural world heritage sites is therefore used as the reference for the national heritage management systems established in the examined herein GCC countries.

### 1972 World Heritage Convention

The provisions of the 1972 Convention are of a general character, as is the case in public international law, and relate to the obligations of States Parties rather than to the local level at which the World Heritage properties are managed.

Nevertheless, it is noteworthy to recall the preamble of the Convention, stating that: ‘(…) Parts of the cultural or natural heritage are of outstanding interest and therefore need to be preserved as part of the world heritage of mankind as a whole (…)’ and ‘(…) that deterioration or disappearance of any item of the cultural or natural heritage constitutes a harmful impoverishment of the heritage of all the nations of the world (…).’

Chapter II of the 1972 Convention, and in particular Articles 4 and 5 are most relevant for establishing obligations of the States toward the cultural and natural heritage on their territory. Art. 4 recognises the duty of States of ensuring the identification protection, conservation, presentation and transmission to future generations of cultural and natural heritage referred to in Art. 1 and 2. Finally, Art. 5 provides a set of minimal basic actions and measures that each State should undertake for the protection, conservation and presentation of the cultural heritage on its territory (Carducci 2008). Among these requirements is the adoption of policy, integration of heritage into comprehensive planning programmes, setting up- well staffed and funded heritage protection authorities, taking the appropriate legal, scientific, technical, administrative and financial measures necessary for the identification, protection, conservation, presentation and rehabilitation of heritage and lastly ensuring the development of research, funding, conservation and other issues.

### Operational Guidelines

The implementation of the 1972 Convention is facilitated through compliance with the provisions of the Operational Guidelines for the Implementation of the World Heritage Convention (UNESCO 2021). This document is regularly updated, usually every 2 years, by the World Heritage Committee to reflect new ideas, knowledge and experiences. Even though the Operational Guidelines are not part of the legal system in any of the examined countries, compliance with its provisions can be requested by the Committee via its decisions and the cases of non-compliance are reported in the process of Reactive Monitoring which in an extreme situation may result with inscribing the property on the List of World Heritage in Danger.^3^

Where heritage management is concerned, Paragraphs 108–112, as well as 118bis and 172 are the most relevant in the Operational Guidelines. According to these provisions, each nominated property should have an appropriate management plan or other documented management system (Para. 108). An effective management system depends on the type, characteristics and needs of the nominated property (Para. 110). Effective management includes a thorough shared understanding of the property; mechanisms for involvement and coordination of the various activities between different partners and stakeholders (Para. 111); a cycle of short, medium and long-term actions to protect, conserve and present nominated property; an integrated approach to planning and management going beyond the property to include any buffer zone as well as broader setting (Para. 112).

Paragraphs 118bis and 172 of the Operational Guidelines are often recalled in the decisions of the World Heritage Committee. They specifically address the issue of development projects that may have an adverse impact on the Outstanding Universal Value, undertaken within the property boundaries, in the buffer zone or in the immediate and wider setting of the World Heritage property.

According to Paragraph 118bis Heritage Impact Assessment^4^ is to be carried out as a pre-requisite for development projects and activities that are planned for implementation within or around a World Heritage property; it should serve to identify development alternatives, potential positive and negative impacts and to recommend mitigation measures against degradation or other negative impacts on the cultural or natural heritage within the property or its wider setting, thus ensuring the long-term safeguarding of the Outstanding Universal Value and the strengthening of heritage resilience.

Last but not least, the paragraph 172 of Operational Guidelines, stipulates the obligation of the States to inform the World Heritage Committee about the plans of major restorations or development projects in an area protected under the Convention. This information should be provided before making any decisions that would be difficult to reverse. Paragraph 172 is an essential element of the previously mentioned Reactive Monitoring mechanism (Paragraphs 169 to 191), and within its framework the missions of experts from the World Heritage Centre and the Advisory Bodies may be invited to the World Heritage site to verify existence of challenging situations and to provide advice to the States.

The language of the Guidelines is highly technical and detailed. It leaves no doubt as to the purposes and measures that each State is to take to ensure the protection, management and presentation of the World Heritage sites.

### Historic Urban Landscapes

2011 UNESCO Recommendation on Historic Urban Landscapes is a document often recalled by the World Heritage Committee in relation to the aspects of the management of historic cities and urban heritage in the broad sense.^5^ During the ten-year period since its adoption, a significant effort was made to establish the framework for implementation of the Recommendation. Considering the text of the Recommendation on Historic Urban Landscapes,^6^ the 2016 HUL Guidebook (WHITRAP et al. 2016) as well as the academic research (Pereira Roders 2019), implementation of the Historic Urban Landscape approach in the cultural heritage law is achieved through addressing the following three main themes:

1. Holistic understanding of heritage and its identification;
2. Integrated management and reference to other laws;
3. Collaborative planning procedures and impact assessment.

The first of the themes is self-explanatory and refers to the identification of significant elements corresponding to different heritage categories and their further protection by listing in the appropriate heritage inventory as well as establishing the site boundaries, buffer zones and, where appropriate, equipping the sites with appropriate fencing and signage. Every legal system examined in the second part of this paper fulfils these requirements to some extent.

The second and third themes which consider the integrated management and the collaborative planning on the other hand are implemented with significant diversity, depending on the policies and priorities of a particular State, balance of conservation and development as well as the position of cultural heritage authority within the government.

The aforementioned standard-setting documents and the particular articles and paragraphs constitute a cohesive regulatory network of reference to the management of the World Heritage sites.

### Heritage Impact Assessment

Heritage Impact Assessment (HIA) is a mechanism of due diligence that is applied to verify the potential negative (or positive) effects of the development project^7^ on cultural heritage receptors (such as the attributes of Outstanding Universal Value of a World Heritage Site) and recommend mitigation measures which allow the project to continue without causing any damage or reducing it to an acceptable level. The HIA should be undertaken in the initial planning stages, prior to the final design and final approval, to enable informed decision making and to guide the collaborative project design (Rodwell and Turner 2018).

In particular, the reconstruction and adaptive use projects should be proposed based on the results of a HIA and the final approval at national level may have to be postponed until a positive opinion from the WH Committee, according to the procedure in the Operational Guidelines mentioned earlier. Therefore, project planning and decision-making should allow enough time to review the project proposal among the stakeholders and interested parties.

In fact, the Heritage Impact Assessment process can be very complicated and take considerable amount of time, especially if the World Heritage Committee is required to approve the project at its annual session.^8^

The HIA should ideally be conducted in line with 2011 ICOMOS Guidelines (under revision) and should take into consideration any sort of impact on the attributes of the OUV. Typically, the considered effects are such as the visual, noise, vibrations, pollution, odor, traffic volume, siltation, visitor’s experience, socio-economic issues and impact on the associated values and wider setting (Wosiński 2017). These effects can also be direct and indirect, temporary or permanent and cumulative with impacts of other projects (ICOMOS 2011).

Impact on heritage should be assessed by a competent, interdisciplinary panel of experts. After conducting preliminary research and familiarising themselves with the characteristics of a given project, experts will evaluate whether and how the Outstanding Universal Value attributes may be destroyed, degraded, obscured or otherwise irreversibly and unfavorably altered. It may also turn out that the project will have only positive effects and will affect not only the quality of life and workplace, but also improve the mobility, accessibility and interpretation of the World Heritage site for groups of residents who previously had limited access to heritage.

The change brought by the development project can be positive or detrimental to the Outstanding Universal Value of the site, and therefore its potential impact and mitigation measures should be carefully examined before the final decision is given about the approval of such project.

### Elements of the adequate heritage management system

Managing cultural World Heritage sites is complex (UNESCO et al. 2013). First and foremost, the actions undertaken must always support the overall goal which is to protect the Outstanding Universal Value and to pass heritage to the next generations.

Secondly, it should be stressed that every site is different, there are different institutions involved in their management and together with the owners and conservation authorities create a very specific system (Ringbeck 2018). For instance, the management of archaeological sites differs significantly from managing urban heritage in the historic cities. In the former, there is usually less stakeholders involved on a daily basis and only involved when planning or responding to project proposal. On the other hand, the needs can change seasonally thorough the year during the excavation campaigns or when conserving the excavated remains. In case of historic cities inscribed on the world heritage list (e.g. Jeddah in Saudi Arabia, Muharraq in Bahrain), there is a number of projects, issues, proposals, initiatives arriving on a daily basis and they have to be addressed timely and respectfully to the Outstanding Universal Value.

Effective heritage management requires understanding the peculiarities existing in each site and managing it with active involvement of all those who have official and traditional mandate (the later one refers to institutions, groups or individuals who are customary entitled to access and care for the place, such as traditional custodians, religious institutions). Depending on the administrative structure of the state, many issues belong to the responsibility of the city administration, other to the regional level and the governmental level.

The minimum management measures are establishing clear boundaries, drawing detailed maps, and designating significant remains, structures, buildings, ensembles as well as urban areas in the monument’s inventory (register, schedule). The way the particular elements of the site are listed in the inventory, enables higher or lower degree of protection by the conservation authorities.

For example, designating every single significant building in a historic district will protect them from being demolished and changed, but will not prevent modifications to the streets, visual corridors, and may not prevent constructing new buildings nearby with conflicting architectural character, incompatible size or materials used. On the other hand, integrating the inventory with GIS and sharing the database with the stakeholders of the planning process can greatly reduce the chances of the designated sites to be negatively affected by the development projects in the future.

In case of World Heritage sites, ensuring protection of all attributes of the OUV with their authenticity and integrity, whether in the context of archaeological sites or historic cities, oftenrequires applying the areal protection for the entirety of the site as well as listing the urban area, with not only the buildings, but also their distinctive street pattern, green areas, courtyards, recreational spaces and other coherent elements. This approach can also be helpful to protect the wider setting of the World Heritage site, for example within the buffer zone.

Review of the State of Conservation reports and Reactive Monitoring mission reports in the examined countries reveals that destruction of cultural heritage is usually an effect of inadequate information exchange, improper planning and coordination, as well as consequence of budget allocation. The sites are therefore not damaged due to a purposeful action targeting heritage but due to the inappropriate design drawings and project tender documentation caused by inadequate planning procedures, lack of due diligence and inter-department consultation as well as the lack of impact assessment. It is therefore of utmost importance to ensure that the information about the location of heritage sites and buildings is known to the planning authorities and thus considered during the earliest planning stages.

Major challenges to heritage places also arrive from large investment opportunities which enable budgetary resources to be spend within relatively short period of time (Ricca 2018). Especially in cases when the funding for the highways, railways and other infrastructure is sponsored by foreign donors without established heritage compliance methods (such as e.g. the International Finance Corporation [IFC] Performance Standard 8), it might be difficult to influence the change of project on the later stage, if there was no opportunity to give feedback during the pre-design stage. For instance, funding for the infrastructure projects in Bahrain from the GCC donors is often approved before the planning procedures are completed or in some cases even initiated, and the transfer of budgeted resources requires fast approval with limited consultations. To prevent this from happening, even with projects of strategic importance, there should be established statutory approval procedures, providing enough time for meaningful assessment of the situation by the authorities. Ideally, the project financing would be conditional and subject to adhering to the international standards and impact assessment studies. In case of lack of procedures or in case the procedures provide only for consultation and not approval or disapproval, heritage protection authorities must continuously exchange information with other governmental agencies as well as regional and city level authorities.

## Part II. Cultural Heritage Protection Laws in the GCC countries

### Overview

There are notable differences between the heritage laws in the GCC countries. In particular, there is a huge time difference between the dates of issue of the principal heritage legislation. The earliest still in force law, Decree No. 11 of 1960 of the Law of Antiquities in Kuwait is from 1960, whilst the latest Law No. 4 of 2020 on Cultural Heritage of Sharjah is from 2020, making the total of 60 years between them. Countries with older law on protection of cultural heritage include Bahrain (1995), Kuwait (1960), and Qatar (1980). The earlier laws issued in the 20th century reflect the predominant thinking of their time about the cultural heritage as tangible cultural property, designated as monuments clearly separated from their setting. (Wosiński 2017, 135) Respectively Oman (2019), Saudi Arabia (2014) and Emirate of Sharjah in the United Arab Emirates (2020) have issued new laws in the last few years. These 21st century laws have been influenced by more matured and integrated heritage theory and practice, codified to certain extend, by the entry into force of three UNESCO Conventions of 2001,^9^ 2003^10^ and 2005^11^ as well as continuously broadening scope of the Operational Guidelines and World Heritage Committee decisions. However, ratification of a complete assortment of UNESCO cultural Conventions isn’t prerequisite to issuing a contemporary heritage law. Among the Conventions adopted in the 21st Century only the 2003 Intangible Heritage Convention has been universally ratified in the GCC (Table 1).

**Table 1:** Cultural Heritage Conventions ratified or acceded by the GCC countries

Cultural Heritage Conventions ratified or acceded by the GCC countries	
Name of the Convention	Country and year of ratification or accession
1954 Convention for the Protection of Cultural Property in the Event of Armed Conflict with Regulations for the Execution of the Convention	Kuwait, 1969 Saudi Arabia, 1971 Qatar, 1973 Oman, 1977 Bahrain, 2008
First Protocol to the Hague Convention of 1954 for the Protection of Cultural Property in the Event of Armed Conflict	Kuwait, 1970 Saudi Arabia, 2007 Bahrain, 2008
Second Protocol to the Hague Convention of 1954 for the Protection of Cultural Property in the Event of Armed Conflict	Qatar, 2000 Bahrain, 2008 Oman, 2011
1970 Convention on the Means of Prohibiting and Preventing the Illicit Import, Export and Transfer of Ownership of Cultural Property	Saudi Arabia, 1976 Qatar, 1977 Oman, 1978 Bahrain, 2014 United Arab Emirates, 2017
1972 Convention concerning the Protection of the World Cultural and Natural Heritage	Saudi Arabia, 1978 Oman, 1981 Qatar, 1984 Bahrain, 1991 United Arab Emirates, 2001 Kuwait, 2002
2001 Convention on the Protection of the Underwater Cultural Heritage	Bahrain, 2014 Saudi Arabia, 2015 Kuwait, 2017
2003 Convention for the Safeguarding of the Intangible Cultural Heritage	Oman, 2005 United Arab Emirates, 2005 Saudi Arabia, 2008 Qatar, 2008 Bahrain, 2014 Kuwait, 2015
2005 Convention on the Protection and Promotion of the Diversity of Cultural Expressions	Kuwait, 2007 Oman, 2007 Qatar, 2009 United Arab Emirates, 2012

Before going further with the analysis of the different laws, it is important to point out that in some Arab States there are two principal cultural heritage laws, one on antiquities and the other on urban heritage.^12^ This approach is not yet common in the GCC, as there does not seem to be any dedicated statutory regulation for the urban heritage of the highest level, except for some municipal regulations on the Emirate level in the UAE^13^ and a chapter in the antiquities law of Saudi Arabia. Protection of urban heritage is somewhat touched by the building construction law and zoning regulations, but only as far as to indicate the need of obtaining no objection from cultural authority in case of urban planning or other projects.

The following sub-chapters are presented in the chronological order, following the year of issue of the principal cultural heritage protection Act in the analysed GCC countries (Table 2).

**Table 2:** The heritage protection laws in Gulf Cooperation Countries

Legally binding principal cultural heritage law		
Country	Year of issue	Full name
Kuwait	1960	Princely Decree No. 11 of 1960 of the Law of Antiquities, amended by Law 9 of 1994 Establishing the Council for Culture, Arts and Letters.
Qatar	1980	Law No. 2 of 1980 on Antiquities. *(Draft new law is under review)*
Bahrain	1995	Decree-Law No 11 of 1995 for the Protection of Antiquities.
Saudi Arabia	2014	Royal Decree No 9/M of 2014 issuing Law of Antiquities, Museums and Urban Heritage.
Oman	2019	Cultural Heritage Law promulgated by Royal Decree 35/2019, amended by the Royal Decree 41/2020.
United Arab Emirates	2017 Federal Law 2020 Sharjah	Federal Law No. 11 of 2017 on Antiquities; Law No. 4 of 2020 on Cultural Heritage of Sharjah.

### Kuwait

Princely Decree No. 11 of 1960 Law of Antiquities, amended by Law No. 9 of 1994 defines that anything made, produced or built by man forty calendar years ago can be considered a monument (Article 3) and that these are either immovable or movable (Article 4).

Immovable monuments are listed in the antiquities register (Article 10 and movable in the record of movable antiquities (Articles 18 and 19).

It is strictly forbidden to damage, injure, disfigure by writing or engraving, transform, detach a part of or falsify the monuments (Article 8). It is also prohibited to alter, repair or move recorded movable antiquities from one place to another, without the authorisation (Article 23).

Owners are obliged to obtain permit prior to any repair or maintenance works on the monuments (Article 13). License is required prior to the construction of any new buildings adjacent to or annexed to a site of antiquities or registered historical building (Article 14).

Urban planning projects are to be consulted with the cultural authority, the new structures should be harmonious with the existing historical environment and protection distance around the historic buildings should be provided (Article 15).

The law foresees penalties for intentional damage to a recorded movable property; destruction of and trespassing on archaeological site or historic building (Article 42). Failure to inform the authorities of the discovery of antiquities; conducting archaeological excavations, trading in antiquities or exporting antiquities without an appropriate license is sanctioned (Article 43).

Finally, counterfeiting movable antiquities; defacing as well as affixing placards or sign-boards on the historic buildings and sites; acquiring unrecorded antiquities and importing antiquities from outside Kuwait is prohibited and sanctioned (Article 44).

### Qatar

The elaboration of the new cultural heritage law in Qatar is still undergoing, therefore at the time of writing of this paper the Law No. 2 of 1980 on Antiquities is still in force and serves as the basis for analysis. The mentioned law defines the monument in Article 1 and, as it the case in previously described country, distinguishes immovable and movable antiquities (Article 2). The immovable antiquities “include the ruins of cities, buildings, archaeological mounts, caves, valleys, fortresses, fences, citadels, religious buildings, schools and others; whether above or below ground or in territorial waters.”

The main authority dealing with heritage matters in Qatar is the Qatar Museum Authority (QMA) which is in charge of assessing the value and significance of the antiquities (Article 3) and is responsible for the registration, maintenance, preservation and search for the antiquities (Article 4). Registration procedure of movable or immovable monuments and its content is defined in Paragraphs 1, 2 and 3 of the Article 4. It is noteworthy, that the law foresees a powerful protection mechanism, according to which the areas surrounding the archaeological sites can also be registered (Article 9), which allows for establishment of the protective buffers (exclusion zones) helpful in preservation of the authentic setting of the sites.

It is prohibited to damage and deform the antiquities as well as to post the signboards or other advertising materials on registered archaeological sites and historical buildings. Permission of the QMA is required for the construction works on the archaeological sites or sampling of soils or any other debris, materials or plants from such sites. No new buildings may be added to any archaeological monument, and no opening or other form of access may be made in archaeological buildings and archaeological fences (Article 7).

Prior approval and supervision of the QMA is required for demolition, partial or total shifting, renovation or restoration of antiquities. It is prohibited to use the registered antiquity locations to store remains or wastes, or as a cemetery. Moreover, no irrigation systems or excavation are permitted in such locations. Approval of QMA is even required for planting or cutting the trees in the protected areas (Article 12). Violations of these provisions are sanctioned by the Article 40, without prejudice to any greater penalty provided in any other law.

The law does not reflect in detail on the protection of urban heritage. It states briefly that urban and rural planning projects must consider the preservation of archaeological sites and landmarks. Also, what’s of an utmost importance is that the approval of QMA is required for the planning projects in areas with confirmed presence of the antiquities (Article 17). However, as the current law does not distinguish categories of urban heritage, historic cities or even the ensembles of buildings, there is no mechanism of protecting these other than designating single buildings.

### Bahrain

Decree-Law No. 11 of 1995 on the Protection of Antiquities introduces the definition of the monument (Article 2) and categorises them as immovable or movable monuments (Article 3).

Immovable monuments comprise the “antiquities attached to the ground, such as archaeological mounds; remains of settlements and burial grounds; fortresses and bastions, historic houses and buildings; pools and qanats, religious buildings such as temples, mosques and others whether on ground or beneath it or in the territorial sea.” Immovable and movable monuments are to be listed in the register of monuments by a decision of the President of Bahrain Authority for Culture and Antiquities (Article 26).

Urban planning projects, such as town or village planning, expansion, beautification or land subdivision in the places where monuments are located require approval of the cultural authority (Article 7). Building and restoration projects nearby archaeological sites or historic buildings require approval as well (Article 8).

Restoration projects of the mosques, on the other hand, require coordination with and approval of the Islamic Affairs and Waqf (Article 8) as the mosques are in the ownership of the religious endowment. There are two Islamic Waqf in Bahrain, one for the Sunni and one for the Shia populations, therefore consultation is required with the relevant Waqf having the ownership over the building in question.

In Bahrain, there is no law specific for built heritage and the regulations of the Decree-Law 11 of 1995 do not cover much of this matter in a comprehensive manner. The regulatory system is supplemented by some provisions of the building construction law and in particular the zoning regulations which foresee some heritage-specific zones as well as building exclusion zones and zones under study.

Destruction of monuments, their alteration, damage or deformation is prohibited by law (Article 6) and sanctioned. Chapter VI of Bahraini Antiquities law introduces penalties for smuggling antiquities outside the country (Article 47); excavating without a license, demolishing, damaging, destroying, deforming, changing the features or removing a part of monument (Article 48); illegal trade in antiquities, forgery, failure to report on the chance discovery, falsifying data or declining to deliver found cultural property to the authorities (Article 42).

### Saudi Arabia

Royal Decree No. 9/M of 2014 issuing Law of Antiquities, Museums and Urban Heritage defines in Article 1 several categories of heritage, including immovable antiquities, movable antiquities, urban heritage, historical sites and folk heritage sites. The definition of the urban heritage describes it as any “man-built creations, including cities, villages, neighborhoods, or buildings inclusive of any spaces, structures and pieces with architectural, historical, scientific, cultural or national value.” This relatively broad legal definition of bult heritage is a significant step forward when compared with other laws in the region.

According to the law, there are two types of heritage inventories: the antiquities record and the urban heritage record. The first one is used to designate movable and immovable antiquities, historical sites, folk heritage sites and folk heritage artifacts (Article 8). The second register, according to the Article 45, is used to designate urban areas and buildings as well as the natural areas surrounding them, such as gardens and green areas. It is noteworthy, that the urban areas are defined in more detail to what was stated in Article 1 and include the “areas consisting of several buildings, forming an urban fabric of cities, villages and neighborhoods (…) as well as surrounding areas necessary for their protection, display or use.

All sites registered to one of the inventories are classified according to their significance level in three categories: A, B and C, corresponding respectively to high, moderate and low significance (Article 25, 45).

It is prohibited to trespass, distort, remove, damage, deface, alter or obliterate the features of the archaeological and urban heritage sites. Placing banners or advertisements on the sites requires prior approval of the Ministry of Culture - hereinafter MOC (Article 6). Severe sanctions for the violation of the antiquities, museums and urban heritage law are included in the Chapter eight (Articles 71–85).

Saudi law requires that the archaeological and urban heritage sites are preserved when planning expansion or improvement of urban or rural areas. Approval of MOC is required for planning of projects on or near the registered sites as well as for construction or renovation permits in areas adjacent to archaeological and urban heritage sites. MOC in coordination with the Ministry of Municipalities and Rural Affairs may also designate construction-free areas (Article 9).

The law also specifies the procedures of coordination with the MOC in numerous cases, e.g. from the Ministry of the Environment, Water and Agriculture and the Ministry of Transportation at the early stage of planning their field projects; the Ministry of Energy, Industry and Mineral Resources before the issue of mining and quarry permits and designation of quarry sites. Ministry of Municipalities and Rural Affairs, as well as other competent government and private entities must notify MOC in case of discovery of antiquities or urban heritage sites during their construction projects, (Article 12).

Provision of high significance and potential protective power is the establishment of a protection zone for the classified urban heritage, archaeological site or building in accordance with the Article 46. The urban heritage protection zone is established “in cities and villages if they have historical, cultural, artistic or scientific significance justifying the preservation. The typical radius of the zone is two hundred meters but can be extended or reduced based on the decision of the MOC.

MOC prepares an urban heritage protection and development plan, in coordination with the Ministry of Municipalities and Rural Affairs and relevant agencies, as part of a comprehensive protection and development program for the area. The plan identifies classified buildings and sites, protection guidelines and requirements, easements, and guidelines for interventions such as: restoration, urban rehabilitation, urban renewal, building codes, land use controls, construction methods, permitted heights, transportation plans, traffic, services and other (Article 47).

Antiquities, Museums and Urban Heritage Fund is established to cover the expenses of the protection, preservation, maintenance, display and utilisation of antiquities and urban heritage. As well as for establishing new museums and to provide support to the private sector, whether individuals, associations or societies, to realise the objectives set out in this Law (Article 20).

The law also foresees incentives for: assisting in detecting an antiquity; providing information of any violation of this law; chance discovery of a significant movable or immovable antiquity or assistance in such discovery and finally contributes to the preservation and prevention of damage to urban heritage and antiquities (Article 89).

### Oman

Cultural Heritage Law promulgated by Royal Decree 35/2019, amended by the Royal Decree 41/2020 and Royal Decree 91/2020, is one of the newest heritage laws in the GCC region, and as such is characterised by much more developed and comprehensive set of provisions. The law stipulates, among others, the legal definitions of: immovable cultural heritage, movable cultural heritage, underwater cultural heritage, intangible cultural heritage, monument, heritage building, heritage complexes, cultural heritage site as well as protective areas (Article 1). Concerning built heritage, the law specifically defines the heritage building as “the building of cultural heritage importance, whether complete or incomplete, which dates back to 100 years” as well as cultural heritage complexes “buildings of cultural heritage importance, due to their architectural design, homogeneity or place in the natural or cultural landscape, whether they are separate or connected to each other.”

The Ministry of Heritage and Tourism establishes the Omani Cultural Heritage Register (Article 32) which includes entries of the heritage assets meeting one or more of the eight value categories (Article 33) and further divides them into grades 1, 2 and 3 (Article 34).

As one of only a few in the examined countries, the Omani law explicitly refers to the World Heritage in several locations, i.e. in the legal definitions (Article 1); matters of coordination of protections efforts, management of sites as well as implementation of decisions and guidelines of the 1972 Convention (Article 5).

The Ministry of Heritage and Tourism is the authority in charge of all matters of cultural heritage such as the management and development of all archaeological, historical, and cultural sites and projects (Article 6).

Development projects or projects related to urban planning may be approved only after coordination with the Ministry (Article 13) and any action related to cultural heritage shall not have a legal effect unless it is in a written form (Article 14).

Similar to the other GCC regulations, it is prohibited to damage or alter any cultural heritage; make acts or statements against Omani heritage; distort, misrepresent, abuse, or exploitation of the intangible cultural heritage; sale, purchase on any material or parts removed from the cultural heritage; dispose of waste in cultural heritage sites (Article 53).

According to the law, a license is required for displaying ads or banners, installing antennas, pipes, signs, planting or cutting trees on the heritage sites and their protected areas; using the material remaining after archaeological excavation (such as sand or rocks); carry out construction works, plantation or plowing in the heritage sites or their protection areas. (Article 54). License is also required for selling, buying, exporting or importing cultural heritage; reproduction of cultural heritage or tradition; establishment of museums or private Heritage houses (Article 16).

The Ministry has full discretion in deciding to stop or remove any activity against cultural heritage and may use the Royal Oman Police to support it in implementing its decisions (Article 60).

It is worth highlighting, that the Omani law foresees the participation of the Ministry in financing the cost of restoration of the cultural heritage places (Article 61).

Last but not least, the law states the penalties for violation of the provisions therein in the Chapter VII of the law (Articles 69–82).

### United Arab Emirates - Emirate of Sharjah

Law No. 4 of 2020 on Cultural Heritage of Sharjah provides the legal definitions of: cultural heritage, material heritage, immovable heritage, movable heritage, intangible cultural heritage, underwater heritage, cultural site and the protected surrounding area (Article 1). The law does not provide a clear definition of built heritage or urban heritage areas. Historic buildings and sites are therefore included under both the immovable material heritage and cultural sites definitions.

The law states that the concerned authority establishes a register of material heritage (Article 30) and a register of intangible heritage (Article 45).

Important regulations regarding built heritage are included under Article 34, according to which in case of urban planning projects, government agencies are obliged to follow the requirements of the concerned authority to preserve the cultural sites and surrounding areas. There is a minimum three-kilometer radius from the cultural sites when planning establishment of industrial projects. Moreover, the concerned authority must approve: planning projects for places containing cultural heritage; settlements near existing or potential cultural sites and last but not least building permits, construction, restoration and maintenance near cultural sites. The concerned authority develops management plan(s) for the cultural sites, in which, among other requirements concerning the assessment of its significance, research plans, documentation and restoration, it should determine the extent of interventions that may occur to it and the social, economic and cultural conditions surrounding it (Article 29).

The Sharjah law refers to international cultural heritage conventions two places. There is a reference to the properties inscribed on the World Heritage List in Article 51 and to the enhanced protection of cultural heritage mechanism in Article 53. Unique among all analysed laws is that the law requires the concerned authority to establish emergency plans and safe places for cultural heritage as well as avoiding establishment of military sites near cultural heritage sites (Article 53).

### Analysis of the 20th Century antiquities laws (old laws)

The 20th Century laws relating to heritage protection are not up to date with the international standard setting treaties, charters and recommendations. The older protection of antiquities laws have not been amended in spite of countries ratifying international conventions since their adoption (see Table 2).

These laws deal only with tangible heritage and refer to it with the vocabulary typically used for ownership and property rights (Fraoua 2012). This fact has a profound repercussions in the management of heritage sites and tendency of acquiring by the State of all designated heritage places in private ownership for the sake of their protection.^14^

The limited number of heritage categories recognised by these laws means that although current legislation allows for proper protection of archaeological sites, the same cannot be said for other kinds of heritage assets. Even where the built heritage is concerned, the provisions are very limited. There is no legal basis to protect historic centres, districts or quarters, ensembles or groups of buildings, cultural landscapes, or natural components connected to the cultural sites.

The older Acts do not contain any provisions regulating the management of sites on their territory inscribed on the World Heritage List (Wosiński 2021). In fact, the implementation of the 1972 Convention is only partial and limited to establishing the relevant authorities and inventories.

The categories of heritage introduced in the ratified International Conventions, such as intangible cultural heritage, cultural landscapes and even urban heritage are not represented in the law. Also lacking is the idea of cultural significance going beyond a tangible monument to include its setting and function.

To sum it up, the old laws refer to the movable cultural property in the language of the 1970 Convention on the Means of Prohibiting and Preventing the Illicit Import, Export and Transfer of Ownership of Cultural Property and to the immovable cultural property in the language of the Venice Charter. It is a property-based system which emphasises the tangible and material characteristics.

The constraints of outdated heritage law in which the authorities in charge of heritage protection and management act, such as the QMA in Qatar or Bahrain Authority for Culture and Antiquities (BACA), don’t stop them from applying better heritage management standards than provided for in their legal systems. The QMA established a well-functioning geolocated digital heritage inventory, initially referred to as Qatar National Historic Environment Record (QNHER) and currently Qatar Cultural Heritage Information Management System (QCHIMS) accessible to staff and stakeholders, greatly facilitating integrated planning in Doha and rest of Qatar (Beardmore et al. 2010).^15^ In case of Bahrain, BACA utilises the possibilities and soft power of the 1972 Convention to the highest possible extent (Wosiński 2021). Inscribing the three most significant heritage sites on the World Heritage List allows BACA to seek support of the World Heritage Centre in case of strategic development projects which would normally be difficult to object on the national level.^16^

### Analysis of the 21st Century antiquities laws (new laws)

The first difference in the new cultural heritage laws in the GCC countries is contained in the very name, which is no longer law on antiquities, but on cultural heritage.

It indicates a shift from the cultural property paradigm and that the newer laws started speaking with the language of values. The tangible paradigm has been enhanced by the parallel intangible heritage system and additional categories of heritage, such as the underwater heritage and heritage sites have their legal definitions.

Noteworthy is the existence of not only no objection procedures and consultation in case of urban planning, but much more comprehensive involvement of cultural heritage authorities in the planning procedures. This follows the advice of 1972 UNESCO Recommendation concerning the Protection at National Level of the Cultural and Natural Heritage and 2011 Recommendation on Historic Urban Landscapes.

A major change has been the introduction of the concepts of heritage site and urban heritage sites. The latter one enables the possibility of listing areas beyond the individual building, which reflects the conservation doctrine of the Washington Charter. Thank to this shift, the streets, courtyards, squares, public spaces as well as the other spaces between the buildings or in their setting can be protected.

The new laws also contain expressis verbis the reference to the World Heritage List, allow creation of legally enforceable buffer zones (exclusion zones) and in some cases refer to the conservation plans. There are also explicitly mentioned references to the urban planning regulations, municipal organs, police and other state services.

Whilst these laws provide additional tools allowing better protection and integration, the successful management under the HUL paradigm requires further normative and non-normative measures. For instance, heritage impact assessment should ideally find its place in the heritage law and be required in case of projects which might have potential negative impact on cultural heritage. Existing regulations under environmental law typically foresee impact assessments in case of large-scale industrial and infrastructural projects, which leaves the “smaller” commercial or building projects unexamined. Saudi Arabia and United Arab Emirates are examples of countries where the Environmental Impact Assessments, required for many development projects every year include assessments of the effects on cultural heritage.^17^ Noteworthy is the example of NEOM project in Saudi Arabia, where compliance with the national environmental law and international environmental standards (e.g. Equator Principles, IFC Performance Standards) is prerequisite to initiating major development projects.^18^

It should be clearly mentioned, that whilst stronger law provides additional tools that can be utilised for the benefit of heritage protection, even the strongest law will not help if it is not implemented and enforced. On the contrary, even the country with the weakest legal framework can protect its heritage in an outstanding way, if there is a strong leadership, experienced team and sufficient implementation of international standards through soft power and executive bylaws.

## Conclusion

All analysed antiquities acts fulfil the baseline requirement of allowing identification of the heritage assets and the protection thereof by listing in the registers. They also allow the acquisition by the state of the antiquities in private ownership. All these laws are successfully used for the preservation of archaeological sites and single standing historic buildings, although protection of the setting of the sites is a challenge.

Analysis of the cultural heritage laws in the previous chapters clearly shows that more recent acts, issued in the 21st century, follow to a certain degree the evolution of the heritage doctrine spearheaded by UNESCO in the framework of the 1972, 2001, 2003 and 2005 Conventions. These countries are less likely to face the challenges of inadequate statutory protection of their cultural heritage and have more measures at their disposal to support integrated and holistic heritage management.

The comprehensive implementation of heritage management measures relies on cohesion and interrelation between the heritage law and other laws related to planning, environmental protection, and impact assessment. The characteristic of the GCC legal systems is however that the planning regulations don’t provide a strong support to protecting cultural heritage sites.

The laws issued after the adoption of UNESCO cultural Conventions from the early 2000’s, show intention of the law-makers for an integrated protection and management of heritage sites and structures, they call for the cross-sectoral consultations, require issuing of no objection certificates and provide approval procedures for the development projects having impact on the protected sites. The management of change in the setting of the protected areas (either World Heritage or nationally listed), especially in the urban areas, remains a challenge in most of the new laws due to lack of areal protection inclusive of all urban elements. Moreover, in case of the urban World Heritage sites, the concept of integrity, as proposed by the Operational Guidelines is difficult to be implemented without the support of the owners of buildings and empty plots within the inscribed area, whose actions are usually economically-driven. The amended legal acts serve as the strong foundation for management of cultural heritage and a starting point for the implementation of the historic urban landscape approach in urban heritage sites.

There is still a considerable way to go before the legal frameworks can be considered comprehensive enough to fulfil all obligations of the States Parties to the 1972 Convention and comply with the heritage management principles in the Operational Guidelines and the HUL Recommendation. The adequate legal protection and management of cultural heritage should allow holistic identification of the heritage values, including all physical and intangible attributes. The GIS information about the protected sites should be shared with the stakeholders of the planning processes through official channels and integrated with the overall zoning and master plan of the city or area. The planning procedures should allow for timely and meaningful feedback from the authority in charge of cultural heritage and allowing modifications to the project design. Finally, the impact assessment should support the system whenever necessary to protect the site from potentially harmful projects.

There is no doubt that great effort has been made and continues to be made by the GCC countries which revised their antiquities laws in the recent years. These States are on the right track toward improved heritage management and their steps should be replicated by the other States with the outdated antiquities laws.

## Data Availability

Not applicable.

## Abbreviations

1972 Convention: 1972 Convention Concerning the Protection of the World Cultural and Natural Heritage
GCC: Gulf Cooperation Countries
HUL: Historic Urban Landscape
ICOMOS: International Council on Monuments and Sites
Operational Guidelines: Operational Guidelines for the Implementation of the World Heritage Convention
OUV: Outstanding Universal Value
QMA: Qatar Museums Authority
UNESCO: United Nations Educational, Scientific and Cultural Organisation
MOC: Ministry of Culture

## Footnotes

1 E.g. decisions concerning the Kingdom of Bahrain: 38 COM 8B.49, 43 COM 7B.42; decisions Concerning Saudi Arabia: 40 COM 7B.27, 42 COM 7B.58; decisions concerning Oman: 40 COM 7B.26, 42 COM 7B.57

2 The draft was shared with the States Parties in March 2021 and the final version will be adopted during the 44th Session of the World Heritage Committee in July 2021.

3 The criteria and procedure for the inscription of properties on the World Heritage List in Danger is regulated in paras. 177–189 of the Operational Guidelines. The same procedure may go one step further and result in the deletion of the site from the World Heritage List altogether, in line with paras. 192–198, for the cases ‘where the property has deteriorated to the extent that it has lost those characteristics which determined its inclusion in the World Heritage List.’

4 Or Environmental Impact Assessment or Strategic Impact Assessment, as appropriate.

5 This Recommendation builds on the foundation established in earlier documents, such as the 1972 UNESCO Recommendation concerning the Protection at National Level of the Cultural and Natural Heritage.

6 Especially Paragraphs 21 to 24 under the Chapter III. Policies and Chapter IV. Tools.

7 Or other kind of change, for example new policy.

8 Due to the global pandemics of Covid-19, the 44th Session of the World Heritage Committee which was scheduled to take place in 2020 was postponed to 2021. As a result, some decisions on approval of the development projects could not take place in the regular schedule which may have resulted with significant delays of the projects on the local level.

9 Convention on the Protection of the Underwater Cultural Heritage.

10 Convention for the Safeguarding of the Intangible Cultural Heritage.

11 Convention on the Protection and Promotion of the Diversity of Cultural Expressions.

12 For example in Jordan there is Law No.21 of 1988 on Antiquities (as amended by Law No. 55 of 2008) and well as Law No. 5 of 2005 on the Protection of Architectural and Urban Heritage

13 Dubai Municipality Resolution No. 1 of 2013 concerning Protection of Historic Buildings and Sites in the Emirate of Dubai.

14 E.g. the Kingdom of Bahrain has an extensive list of land plots recommended for acquisition due to the presence of archaeological heritage or historic buildings.

15 More information is available on the QMA website: https://qm.org.qa/en/about-us/archaeology-and-heritage/cultural-heritage-management/

16 In total approximately 30 individual buildings and sites, thanks to the serial nomination approach.

17 Executive Regulations for Environmental Permits implementing the Royal Decree No. M/165 of 2020 in Saudi Arabia; Resolution No. 37 of 2001 issuing the Environmental Impact Assessment Regulation for installations implementing the Federal Law No 24 of 1999 On the Protection and Development of the Environment in United Arab Emirates.

18 Author was involved in preparation of some of the Environmental and Social Impact Assessment reports in NEOM region in 2021–2022.
